# Stable solution to *l*_2,1_-based robust inductive matrix completion and its application in linking long noncoding RNAs to human diseases

**DOI:** 10.1186/s12920-017-0310-1

**Published:** 2017-12-28

**Authors:** Ashis Kumer Biswas, Dongchul Kim, Mingon Kang, Chris Ding, Jean X. Gao

**Affiliations:** 1 Department of Computer Science and Engineering, University of Colorado Denver, Denver, 80204 Colorado USA; 2 Department of Computer Science and Engineering, University of Texas at Arlington, Arlington, 76019 Texas USA; 3 Department of Computer Science, University of Rio Grande Valley, Edinburg, 78541 Texas USA; 4 Department of Computer Science, Kennesaw State University, Marietta, 30060 Georgia USA

**Keywords:** Matrix completion, Inductive learning, Long noncoding RNA, Human disease phenotypes, Association inference

## Abstract

**Backgrounds:**

A large number of long intergenic non-coding RNAs (lincRNAs) are linked to a broad spectrum of human diseases. The disease association with many other lincRNAs still remain as puzzle. Validation of such links between the two entities through biological experiments are expensive. However, a plethora lincRNA-data are available now, thanks to the High Throughput Sequencing (HTS) platforms, Genome Wide Association Studies (GWAS), etc, which opens the opportunity for cutting-edge machine learning and data mining approaches to extract meaningful relationships among lincRNAs and diseases. However, there are only a few *in silico* lincRNA-disease association inference tools available to date, and none of them utilizes side information of both the entities simultaneously in a single framework.

**Methods:**

The recently developed Inductive Matrix Completion (IMC) technique provides a recommendation platform among two entities considering respective side information about them. However, the formulation of IMC is incapable of handling noise and outliers that may be present in the datasets, while data sparsity consideration is another issue with the standard IMC method. Thus, a robust version of IMC is needed that can solve the two issues. As a remedy, in this paper, we propose Stable Robust Inductive Matrix Completion (SRIMC) that utilizes the *l*_2,1_ norm based regularization to optimize the objective function with a unique 2-step stable solution approach.

**Results:**

We applied SRIMC to the available association data between human lincRNAs and OMIM disease phenotypes as well as a diverse set of side information about the lincRNAs and the diseases. The method performs better than the state-of-the-art methods in terms of *p* *r* *e* *c* *i* *s* *i* *o* *n* *@* *k* and *r* *e* *c* *a* *l* *l* *@* *k* at the top-*k* disease prioritization to the subject lincRNAs. We also demonstrate that SRIMC is equally effective for querying about novel lincRNAs, as well as predicting rank of a newly known disease for a set of well-characterized lincRNAs.

**Conclusions:**

With the experimental results and computational evaluation, we show that SRIMC is robust in handling datasets with noise and outliers as well as dealing with novel lincRNAs and disease phenotypes.

## Background

### LincRNA-disease association inference problem

It is a surprising fact that, only 2% of the entire human genome codes for proteins [1]. In recent years, it has become evident that the non-protein coding portion of the genome, especially the long intergenic non-coding RNAs (lincRNAs) having length more than 200 bases each with no overlaps with any annotated protein-coding regions, are of critical functional importance. These lincRNAs demonstrate diverse molecular mechanisms and implicate various human diseases [2]. With the advent of the high-throughput genomic technologies, a large number of lincRNAs have been cataloged [3]. However, fully annotating the functions of the lincRNAs and their involvements in human disease implications still remain a challenge for the researchers. Developing machine learning algorithm to rank disease implications by a given lincRNA based on prior knowledge would be beneficial to the community for tackling the challenge.

### Limitations of existing algorithms

There are several long non-coding RNA (lncRNA)-disease association inference tools developed in the past few years. But, there is a small number of tools that actually solved the lincRNA-disease inference problem. Due to the complexities in the relationship and the available datasets, only a small number of experimentally validated associations have been reported in the lncRNAdisease database [4]. Therefore, using multiple complementary data sources in the algorithm is important to predict potential lincRNA and disease associations. For example, LRLSLDA [5], K-RWRH [6], and TslncRNA-disease [7] belong to a family of network based association identification methods. Each of the algorithms use biological networks, such as lincRNA similarity network and disease similarity network to develop their prediction model. Then by using the model they infer lincRNA-disease connections by either using random walk procedure on a derived biological network or by computing a similarity measure between nodes with known disease implications. The association inference problem can also be tackled through the use of matrix completion based algorithms; Non-negative Matrix Factorization (NMF) belongs to this family of solution strategy. But, it suffers the cold start problem, due to the inability to address the inference predictions of the diseases for novel lincRNAs and vice versa. Furthermore, these methods were presented on a very small set of associations and developed without considering the scalability (e.g., around 200 lncRNAs compared to more than 8000 lincRNAs available to date from the research by [3] remain overlooked). However, the methods utilizing the lincRNA-expression profiles to build similarity networks dealing with a small number of disease classes. So, they fall short in generalizing their prediction to identify novel diseases-lincRNA connections. Owing to the fact that, a plethora of side information about the lincRNAs and the disease phenotypes are available, and the data is growing extensively every single day. Inductive Matrix Completion (IMC) based algorithms utilize side information about both the lincRNAs and diseases along with the known association evidences to predict missing associations [8]. But, the standard IMC uses the least square error function which is known to be unstable in presence of noise and outliers [9]. A stable and robust version of the IMC is thus needed in this problem.

### Outline of our proposed approach

We propose a novel stable robust formulation of IMC using *ℓ*_2,1_ norm based error function, as well as *ℓ*_2,1_ based regularizer. We call the proposed method “robust” because it can handle noise better than the standard IMC. Also, we call the method “stable” because of the fact that it utilizes a 2-step stable strategies to solve the problem.

### Summary of contributions

- We first describe a Robust IMC approach that introduces *l*_2,1_ norms in both its penalty function and the regularization. We then propose Stable Robust IMC (SRIMC) that can handle outliers and noise in the dataset and also joint sparsity. The solution strategy breaks the problem into two separate and independent problems, where each of the sub-problem has stable solution and easy to compute. Hence, in terms of computational complexity and reliability SRIMC should be a better option.
- We provide an application of our RIMC and SRIMC methods to solve the lincRNA-disease association inference problem. We show that RIMC and SRIMC can perform induction to decipher associations between a novel disease and a novel lincRNA, based on the side information about them we have, that are not provided during learning phase. This is unlike the traditional matrix factorization methods and network-based inference methods discussed earlier which are transductive in nature.
- We demonstrate that the integration of diverse features of the lincRNAs and the diseases available through publicly available data-servers can overcome worse predictive performance issue faced by the inference tools which occurs due to the extreme sparsity inherent to the lincRNA-disease association dataset.
- We present a comparison of our proposed RIMC, SRIMC with standard IMC as well as the state-of-the-art lincRNA-disease association methods.

The rest of the paper is organized as follows. In “Methods” section we propose the robust IMC formulation using *ℓ*_2,1_ norm, underline the advantages of the proposed algorithm compared with the standard IMC as well as standard NMF approaches. Here we also show the correctness of the proposed algorithm. In “Results” section we present the experimental setup and the dataset. In “Discussions” section, we present the results of the experiments, and comparative study on the performance of the proposed algorithm with the base-line methods. Finally, in “Conclusions” section we conclude the paper. A preliminary version of this work has been reported in [10].

## Methods

### Stable robust inductive Matrix completion (SRIMC) strategy

In this section we review standard Inductive Matrix Completion method; then we present our robust IMC (RIMC) formulation. And finally, we present the Stable Robust IMC (SRIMC) algorithm. Later, we provide a computational algorithm for our proposed method along with the correctness of the algorithm.

### Review on standard IMC

The Inductive Matrix Completion approach [8] includes side information of both the row and column entities. The formulation solves the issue “cold-start” problem in a transductive setup (e.g., standard NMF, etc.). Therefore we can predict association between new entities that are not included in the data matrix available during the training time. Let’s consider an association matrix, $A\in \mathbb {R}^{M\times N}$ denoting the links between *M* row entities and *N* column entities. We also have side information of both the row and column entities and the information is encapsulated in two matrices $X\in \mathbb {R}^{M\times m}$ and $Y\in \mathbb {R}^{N\times n}$ containing *m* features of the *M* row entities and *n* features of the *N* column entities respectively. Equation 1 defines the the objective function of the standard IMC.

1 $$\begin{array}{*{20}l} {}\underset{W,H}{\text{min}}&\quad \varphi = \frac{1}{2}\left\|A-XWH^{T}Y^{T}\left\|{~}^{2}_{F} + \frac{\lambda_{1}}{2} \right\|W\left\|{~}^{2}_{F} + \frac{\lambda_{2}}{2}\right\|H\right\|{~}^{2}_{F}&\\ &\text{such that},\quad W\geq 0, H\geq 0,& \end{array} $$

where *λ*_1_,*λ*_2_ are the regularization parameters that weighing between the accrued loss on the observed entries and the trace norm regularization constraints. Here, an entry *A*_*ij*_ is modeled as $\mathbf {x}^{T}_{i}Z\mathbf {y}_{j}$, where $Z\in \mathbb {R}^{m\times n}$ is a low-rank matrix to be recovered by solving Eq. 1. It is solved in a way that *Z* becomes the multiplication of two factor matrices *W* and *H*, that is, *W* *H* ^*T*^, where $W\in \mathbb {R}^{m\times r}$ and $H\in \mathbb {R}^{n\times r}$. Equation 1 can be easily solved using Algorithm 1.



After Algorithm 1 returns, we get the two factor matrices *W* and *H*. These two matrices can be used to compute missing association scores between the row and the column entities. It can also provide prediction score for an association between a known row entity with an new column entity, or a known column entity with a new row entity, or both new row and column entities.

### Robust IMC (RIMC) formulation

One limitation of the standard IMC is that it is prone to outliers in the given dataset. Given $A\in \mathbb {R}^{M\times N}, X\in \mathbb {R}^{M\times m}, Y\in \mathbb {R}^{N\times n}$, the loss function of the standard IMC is:

4 $$ \left\|A - XWH^{T}Y^{T}\left\|{~}^{2}_{F} = \sum_{i=1}^{M}\right\|A_{i,:}-X_{i,:}WH^{T}Y^{T}\right\|{~}_{2}^{2}  $$

Here, a squared residual error gets accumulated in each iteration in the optimization step, meaning only a few outliers may result in large error. Another shortcoming of the the standard IMC is that it can not handle joint sparsity across feature data matrices *X* and *Y*. Therefore, a solution to each of the limitations is needed. The initial hypothesis of RIMC was presented by [10]. The robust IMC, instead of using the *ℓ*_2_ norm based loss function involves *ℓ*_2,1_ norm in defining the loss function which is:

5 $$ \left\|A - XWH^{T}Y^{T}\right\|{~}_{2,1} = \sum_{i=1}^{M}\sqrt{\sum_{j=1}^{N}\left(A-XWH^{T}Y^{T}\right)^{2}_{{ij}}}  $$

Due to the fact that the errors are not squared in each step, the approach has great advantage to handle outliers than that of standard IMC based approaches. The generalized objective function of the RIMC can be stated as:

6 $$\begin{array}{*{20}l} \underset{W,H}{\text{min}} &\quad\varphi = \left\|A-XWH^{T}Y^{T}\right\|{~}_{2,1} + \lambda_{1} R(W) + \lambda_{2} R(H)&\\ &\text{such that},\quad W\geq 0, H\geq 0 &  \end{array} $$

Here, we have several options as the regularization function *R*(·); such as: $R_{1}(B) = ||B||^{2}_{F}$, $R_{2}(B) = \sum _{i=1}^{M}||B_{i,:}||_{1}$, $R_{3}(B) = \sum _{i=1}^{M}||B_{i,:}||^{0}_{2}$ and $R_{4}(B) = \sum _{i=1}^{M}||B_{i,:}||_{2}$. Here, *R*_1_(·) is the ridge regularization and is adapted in the standard IMC formulation, *R*_2_(·) is the LASSO regularization which is a non-convex function and difficult to optimize. *R*_3_(·) involves the *ℓ*_0_ norm and is the most desirable [11], and *R*_4_(·) employs the *ℓ*_2,1_ norm. *R*_4_(·) was chosen because the function is convex and we can easily optimize the objective function involving this kind of regulizer [12].

Thus given the data matrices *A*,*X*,*Y*, in this paper we optimize the following robust IMC formulation:

7 $$\begin{array}{*{20}l} \underset{W,H}{\text{min}} &\quad\varphi = \left\|A-XWH^{T}Y^{T}\right\|{~}_{2,1} + \lambda_{1} \|W\|{~}_{2,1} + \lambda_{2} \|H\|{~}_{2,1}\\ &\text{such that},\quad W\geq 0, H\geq 0 \end{array} $$

### Algorithm for RIMC (version 1)

In order to solve Eq. 7, Algorithm 2 can be adapted [10].



### Correctness of the RIMC Algorithm (version 1)

#### **Theorem 1**

At convergence, the converged solution *W* ^∗^ of the updating rule in Algorithm *2* satisfies the KKT condition.

#### *Proof*

The KKT condition for *W* with constraints *W*_*ik*_≥0, with *i*=1⋯*m*,*k*=1⋯*r* is:

8 $$ \frac{\partial \varphi(W)}{\partial W_{{ik}}}W_{{ik}} = 0, \forall i,k  $$

Now, the partial derivative is

9 $$ {}\begin{aligned} &\frac{\partial \varphi(W)}{\partial W_{{ik}}}=&\\ &\sum_{\alpha=1}^{M}\frac{1}{\sqrt{\sum_{\beta=1}^{N}\left(A-XWH^{T}Y^{T}\right)^{2}_{\alpha\beta}}} \cdot\sum_{\beta^{\prime}=1}^{N}\left(A-XWH^{T}Y^{T}\right)_{\alpha\beta^{\prime}}\cdot&\\ & \frac{\partial}{\partial W_{{ik}}} \left(A-XWH^{T}Y^{T}\right)_{\alpha\beta^{\prime}}+ \lambda_{1}\sum_{\alpha=1}^{m}\frac{1}{\sum_{\beta=1}^{r} W^{2}_{\alpha\beta}}\cdot W_{\alpha\beta}\cdot \frac{\partial W_{\alpha\beta} }{\partial W_{{ik}}}&\\ &=\sum_{\alpha=1}^{M} D_{\alpha\alpha}\sum_{\beta^{\prime}=1}^{N}\left(A-XWH^{T}Y^{T}\right)_{\alpha\beta^{\prime}}\left(-X^{T}e_{M}e^{T}_{N}YH\right)_{{ik}} &\\ & + \lambda_{1}P_{{ii}}W_{{ik}}& \end{aligned}  $$

where $e_{s} = (1,\cdots, 1)^{T}\in \mathbb {R}^{s}$ is a vector with all 1s. Also, $D, P \in \mathbb {R}^{m\times m}$ are the two diagonal matrices with the diagonal elements given by:

10 $$ D_{{ii}} = 1 \bigg/\sqrt{\sum_{j=1}^{N} \left(A-XWH^{T}Y^{T}\right)^{2}_{{ij}}}  $$

11 $$ P_{{ii}} = 1 \bigg/\sqrt{\sum_{j=1}^{r} W^{2}_{{ij}}}  $$

Now, let us continue from Eq. 9:

12 $$ {}\begin{aligned} \frac{\partial \varphi(W)}{\partial W_{{ik}}}&=-\left(e_{m}e^{T}_{M}Ae_{N}e^{T}_{M}De_{M}e^{T}_{m}X^{T}e_{M}e^{T}_{N}YH\right)_{{ik}}\\ &\quad \left(\!e_{m}e^{T}_{M}XWH^{T}Y^{T}e_{N}e^{T}_{M}De_{M}e^{T}_{m}X^{T}e_{M}e^{T}_{N}YH\!\right)_{{ik}}\\ &\quad+ \lambda_{1}P_{{ii}}W_{{ik}} \end{aligned}  $$

Thus, the KKT condition for *W* is:

13 $$\begin{array}{@{}rcl@{}}{} && \left[-\left(e_{m}e^{T}_{M}Ae_{N}e^{T}_{M}De_{M}e^{T}_{m}X^{T}e_{M}e^{T}_{N}YH\right)_{{ik}}\right.\\ {} &&\quad\!\!\! + \left(e_{m}e^{T}_{M}XWH^{T}Y^{T}e_{N}e^{T}_{M}De_{M}e^{T}_{m}X^{T}e_{M}e^{T}_{N}YH\right)_{{ik}} \qquad\\ &&\qquad\qquad \qquad\qquad\qquad\left.{\vphantom{a^{a^{a}}}} + \lambda_{1}P_{{ii}}W_{{ik}}\right]W_{{ik}} =0, \forall i,k  \end{array} $$

But, once *W* converges (according to Algorithm 2), the converged solution (*W* ^∗^) satisfies 
 which can be written as

$$\begin{array}{@{}rcl@{}} && \left[-\left(e_{m}e^{T}_{M}Ae_{N}e^{T}_{M}De_{M}e^{T}_{m}X^{T}e_{M}e^{T}_{N}YH\right)_{{ik}}\right.\\ &&\quad\!\!\! + \left(e_{m}e^{T}_{M}XW^{*}H^{T}Y^{T}e_{N}e^{T}_{M}De_{M}e^{T}_{m}X^{T}e_{M}e^{T}_{N}YH\right)_{{ik}}\\ &&\qquad\qquad\qquad\qquad\qquad\qquad \left.{\vphantom{^{a^{a}}}}+ \lambda_{1}P_{{ii}}W^{*}_{{ik}}\right]W^{*}_{{ik}} =0 \end{array} $$

This is identical to Eq. 43. Thus, the converged solution *W* ^∗^ satisfies the KKT condition. □

#### **Theorem 2**

At convergence, the converged solution *H* ^∗^ of the updating rule in Algorithm *2* satisfies the KKT condition.

#### *Proof*

The KKT condition for *H* with constraints *H*_*jk*_≥0, with *j*=1⋯*n*,*k*=1⋯*r* is:

14 $$ \frac{\partial \varphi(H)}{\partial H_{{jk}}}H_{{jk}} = 0, \forall j,k  $$

Now, the partial derivative is

15 $$ \begin{aligned} &\frac{\partial \varphi(H)}{\partial H_{{jk}}}=&\\ &\sum_{\alpha=1}^{M}\frac{1}{\sqrt{\sum_{\beta=1}^{N}\left(A-XWH^{T}Y^{T}\right)^{2}_{\alpha\beta}}} \cdot\sum_{\beta^{\prime}=1}^{N}\!\left(A-XWH^{T}Y^{T}\right)_{\alpha\beta^{\prime}}\cdot&\\ & \frac{\partial}{\partial H_{{jk}}} \left(A-XWH^{T}Y^{T}\right)_{\alpha\beta^{\prime}}+ \lambda_{2}\sum_{\alpha=1}^{n}\frac{1}{\sum_{\beta=1}^{r} H^{2}_{\alpha\beta}}\cdot H_{\alpha\beta}\cdot \frac{\partial H_{\alpha\beta} }{\partial H_{{ik}}}&\\ &=\sum_{\alpha=1}^{M} D_{\alpha\alpha}\sum_{\beta^{\prime}=1}^{N}\left(A-XWH^{T}Y^{T}\right)_{\alpha\beta^{\prime}}\left(-Y^{T}e_{N}e^{T}_{M}XW\right)_{{jk}} &\\ &+ \lambda_{2}Q_{{jj}}H_{{jk}},& \end{aligned}  $$

where *D* is already defined in Eq. 10, and $Q \in \mathbb {R}^{n\times n}$ is a diagonal matrix with the diagonal elements given by:

16 $$ Q_{{jj}} = 1 \bigg/\sqrt{\sum_{i=1}^{r} H^{2}_{{ji}}}  $$

Now, let us continue from Eq. 15:

17 $$\begin{array}{*{20}l} {}\frac{\partial \varphi(H)}{\partial H_{{jk}}}=&-\left(e_{n}e^{T}_{M}Ae_{N}e^{T}_{M}De_{M}e^{T}_{n}Y^{T}e_{N}e^{T}_{M}XW\right)_{{jk}}\\ &\left(e_{n}e^{T}_{M}XWH^{T}Y^{T}e_{N}e^{T}_{M}De_{M}e^{T}_{n}Y^{T}e_{N}e^{T}_{M}XW\right)_{{jk}}\\& + \lambda_{2}Q_{{jj}}H_{{jk}} \end{array} $$

Thus, the KKT condition for *H* is:

18 $$\begin{array}{*{20}l} &{}\left[-\left(e_{n}e^{T}_{M}Ae_{N}e^{T}_{M}De_{M}e^{T}_{n}Y^{T}e_{N}e^{T}_{M}XW\right)_{{jk}}\right.\\ &{} \quad\!\!\! + \left(e_{n}e^{T}_{M}XWH^{T}Y^{T}e_{N}e^{T}_{M}De_{M}e^{T}_{n}Y^{T}e_{N}e^{T}_{M}XW\right)_{{jk}}\\ &{}\qquad\qquad\qquad\qquad\qquad\!\! \left.{\vphantom{^{a^{a}}}}+ \lambda_{2}Q_{{jj}}H_{{jk}}\right]H_{{jk}} =0, \forall j,k \end{array} $$

But, once *H* converges (according to Algorithm 2), the converged solution (*H* ^∗^) satisfies 
 which can be written as

19 $$\begin{array}{*{20}l} &{} \left[-\left(e_{n}e^{T}_{M}Ae_{N}e^{T}_{M}De_{M}e^{T}_{n}Y^{T}e_{N}e^{T}_{M}XW\right)_{{jk}}\right.\\ &{} \quad\!\! + \left(e_{n}e^{T}_{M}XWH^{T*}Y^{T}e_{N}e^{T}_{M}De_{M}e^{T}_{n}Y^{T}e_{N}e^{T}_{M}XW\right)_{{jk}}\\ &{}\left.\qquad\qquad\qquad\qquad\qquad\qquad\; + \lambda_{2}Q_{{jj}}H^{*}_{{jk}}\right]H^{*}_{{jk}} =0 \end{array} $$

This is identical to Eq. 47. Thus, the converged solution *H* ^∗^ satisfies the KKT condition. □

### Algorithm for RIMC (version 2)

We can also solve the robust IMC optimization problem (Eq. 7) without the use of the *e* vectors. It is demonstrated in Algorithm 3.



### Convergence of the RIMC Algorithm (version 2)

Here, we present the proof of the convergence of Algorithm 3.

#### **Theorem 3**

Algorithm *3* will monotonically decrease the objective function of the problem (Eq. 7) in each iteration and converge to the global optimum of the problem.

However, it can be rephrased using the following two statements:

(A) Updating *H* using the *H* update equation in Algorithm *3* while fixing *W*, the objective function of the problem (Eq. 7) monotonically decreases.
(B) Updating *W* using the *W* update equation in Algorithm *3* while fixing *H*, the objective function of the problem (Eq. 7) monotonically decreases.

#### *Proof*

We prove Theorem 3 (A, B) separately in the following two sections. □

### Proof of Theorem 3(A): Updating of *H*

#### *Proof*

We now focus on proving Theorem 3(A). The proof requires the following two lemmas: (Lemma 4 and 5). □

#### **Lemma 4**

Let, *H* ^(*t*)^ be the *H* at the *t* ^th^ iteration, and *H* ^(*t*+1)^ is obtained from the next iteration. Then, under the *H* update rule in Algorithm *3*, the following inequality holds.

20 $$ {}\begin{aligned} tr\left(\left(A-XW{H^{(t+1)}}^{T}Y^{T}\right)^{T} D \left(A-XW{H^{(t+1)}}^{T}Y^{T}\right)\right)\\ + \lambda_{1} tr\left(W^{T}PW \right) + \lambda_{2} tr\left({H^{(t+1)}}^{T}QH^{(t+1)}\right)\\ \leq tr\left(\left(A-XW{H^{(t)}}^{T}Y^{T}\right)^{T} D \left(A-XW{H^{(t)}}^{T}Y^{T}\right)\right)\\ + \lambda_{1} tr\left(W^{T}PW \right)+ \lambda_{2} tr\left({H^{(t)}}^{T}QH^{(t)}\right), \end{aligned}  $$

where, $D_{{ii}} = 1 \bigg /\sqrt {\sum _{j=1}^{N} (A-XW{H^{(t)}}^{T}Y^{T})^{2}_{{ij}}}$, and $Q_{{ii}} = 1 \bigg /\sqrt {\sum _{j=1}^{r} H^{{(t)}^{T}}_{{ij}}}$

The proof of Lemma 4 is given in section Proof of Lemma 4.

#### **Lemma 5**

Under the *H* update rule in Algorithm 3, the following inequality holds:

$${}\begin{aligned} & \left\| A-XW{H^{(t+1)}}^{T}Y^{T}\right\|_{2,1} +\lambda_{1}\left\|W\right\|_{2,1}+\lambda_{2}\left\|H^{(t+1)}\right\|_{2,1}&\\ &- \left\|A-XW{H^{(t)}}^{T}Y^{T}\right\|_{2,1} -\lambda_{1}\left\|W\right\|_{2,1}-\lambda_{2}\left\|H^{(t)}\right\|_{2,1}\leq &\\ \end{aligned} $$

21 $$ {}\begin{aligned} &\frac{1}{2}\left\{tr\left(\left(A-XW{H^{(t+1)}}^{T}Y^{T}\right)^{T} D \left(A-XW{H^{(t+1)}}^{T}Y^{T}\right)\right)\right.&\\ & \left.+ \lambda_{1} tr\left(W^{T}PW \right) + \lambda_{2} tr\left({H^{(t+1)}}^{T}QH^{(t+1)}\right)\right.&\\ &\left.- tr\left(\left(A-XW{H^{(t)}}^{T}Y^{T}\right)^{T} D \left(A-XW{H^{(t)}}^{T}Y^{T}\right)\right)\right.&\\ &\left.- \lambda_{1} tr\left(W^{T}PW \right)- \lambda_{2} tr\left({H^{(t)}}^{T}QH^{(t)}\right)\right\},& \end{aligned}  $$

*where* *D*,*P*,*Q* *matrices are defined earlier.*

The proof of Lemma 5 is given in section Proof of Lemma 5.

Now, if we take a look at the right hand side of the inequality in Eq. 21, the value is negative or zero according to Lemma 4. This completes the proof that the objective function of Eq. 7 decreases monotonically.

### Proof of Theorem 3(B): updating of *W*

#### *Proof*

We now focus on proving Theorem 3(B). The proof requires the following two lemmas: (Lemma 6 and 7). □

#### **Lemma 6**

Let, *W* ^(*t*)^ be the *W* at the *t* ^th^ iteration, and *W* ^(*t*+1)^ is obtained from the next iteration. Then, under the *W* update rule in Algorithm *3*, the following inequality holds.

22 $$ {}\begin{aligned} tr\left(\left(A-XW^{(t+1)}{H}^{T}Y^{T}\right)^{T} D \left(A-XW^{(t+1)}{H}^{T}Y^{T}\right)\right)\\ + \lambda_{1} tr\left({W^{(t+1)}}^{T}PW^{(t+1)} \right) + \lambda_{2} tr\left({H}^{T}QH\right)\\ \leq tr\left(\left(A-XW^{(t)}{H}^{T}Y^{T}\right)^{T} D \left(A-XW^{(t)}{H}^{T}Y^{T}\right)\right)\\ + \lambda_{1} tr\left({W^{(t)}}^{T}PW^{(t)} \right)+ \lambda_{2} tr\left({H}^{T}QH\right), \end{aligned}  $$

where, *D*,*P*,*Q* are defined earlier.

Proof of Lemma 6 is provided in section Proof of Lemma 6.

#### **Lemma 7**

Under the *W* update rule in Algorithm *3*, the following inequality holds:

23 $$ {}\begin{aligned} &\left\|A-XW^{(t+1)}{H}^{T}Y^{T}\right\|_{2,1} +\lambda_{1}\left\|W^{(t+1)}\right\|_{2,1}+\lambda_{2}\left\|H\right\|_{2,1}&\\ &- \left\|A-XW^{(t)}{H}^{T}Y^{T}\right\|_{2,1} -\lambda_{1}\left\|W^{(t)}\right\|_{2,1}-\lambda_{2}\left\|H\right\|_{2,1}\leq &\\ &\frac{1}{2}\left\{tr\left(\left(A-XW^{(t+1)}{H}^{T}Y^{T}\right)^{T} D \left(A-XW^{(t+1)}{H}^{T}Y^{T}\right)\right)\right.&\\ & \left.+ \lambda_{1} tr\left({W^{(t+1)}}^{T}PW^{(t+1)} \right) + \lambda_{2} tr\left({H}^{T}QH\right)\right.&\\ &\left.- tr\left(\left(A-XW^{(t)}{H}^{T}Y^{T}\right)^{T} D \left(A-XW^{(t)}{H}^{T}Y^{T}\right)\right)\right.&\\ &\left.- \lambda_{1} tr\left({W^{(t)}}^{T}PW^{(t)} \right)- \lambda_{2} tr\left({H}^{T}QH\right)\right\},& \end{aligned}  $$

where *D*,*P*,*Q* matrices are defined earlier.

Proof of Lemma 7 is provided in section Proof of Lemma 7.

Now, if we take a look at the right hand side of the inequality in Eq. 23, the value is negative or zero according to Lemma 6. This completes the proof that the objective function of Eq. 7 decreases monotonically.

### Proof of Lemma 4

#### *Proof*

We can re-write Eq. 20 as follows:

24 $$\begin{array}{@{}rcl@{}} J\left(H^{(t+1)}\right) \leq J\left(H^{(t)}\right), \end{array} $$

where

25 $$\begin{array}{@{}rcl@{}} J(H) &=& tr\left(A-XWH^{T}Y^{T}\right)^{T}D\left(A-XWH^{T}Y^{T}\right)\\ &&+\lambda_{1} tr\left(W^{T}PW\right) +\lambda_{2} tr\left(H^{T}QH\right) \end{array} $$

And, according to the statement of Lemma 4, under the *H* update rule Algorithm 3, *J*(*H*) monotonically decreases. In order to prove the statement, we follow the approaches utilizing auxiliary functions [13, 14]. □

#### **Definition 1**

*G*(*H*,*H* ^′^) is an auxiliary function for the function *J*(*H*) if *G*(*H*,*H* ^′^)≥*J*(*H*) for all *H* ^′^ and *G*(*H*,*H*)=*J*(*H*).

Now, we define:

$$\begin{array}{@{}rcl@{}} H^{(t+1)} = \underset{H}{\text{argmin}} \quad G\left(H,H^{(t)}\right) \end{array} $$

So, we have

$$\begin{array}{@{}rcl@{}} J\left(H^{(t+1)}\right) &=& G\left(H^{(t+1)},H^{(t+1)}\right) \leq G\left(H^{(t+1)},H^{(t)}\right)\\ &\leq & G\left(H^{(t)},H^{(t)}\right) = J(H^{(t)}) \end{array} $$

This proves that *J*(*H* ^(*t*)^) is monotonically decreasing.

Now the important steps in the remainder of the proof are: (a) determine a proper auxiliary function, and (b) find the global minima of the auxiliary function.

#### **Lemma 8**

The function

26 $$\begin{array}{*{20}l}  G(H,{H}^{\prime}) &= tr\left(A^{T}DA\right) - 2tr\left(YHW^{T}X^{T}DA\right)\\ &\quad +\lambda_{1} tr\left(W^{T}PW\right)+ \lambda_{2} tr\left(H^{T}QH\right) \\ &\quad + \sum_{i=1}^{n}\sum_{j=1}^{r} \frac{\left(Y^{T}YH^{\prime}W^{T}X^{T}DXW\right)_{{ij}}H^{2}_{{ij}}}{{H^{\prime}}_{{ij}}}  \end{array} $$

is an auxiliary function for *J*.

#### *Proof*

Now *J*(*H*) of Eq. 25 can be re-written as:

27 $$\begin{array}{*{20}l} J(H) &= tr(A^{T}DA) - 2tr\left(YHW^{T}X^{T}DA\right)\\ &\quad +\lambda_{1} tr\left(W^{T}PW\right)+ \lambda_{2} tr\left(H^{T}QH\right) \\ &\quad + tr\left(H^{T}Y^{T}YHW^{T}X^{T}DXW\right) \end{array} $$

Now we will be applying the following inequality of matrices according to the investigations by [14, 15]:

28 $$\begin{array}{@{}rcl@{}} tr\left(H^{T}\Lambda HB\right) \leq \sum_{i}\sum_{j}\left(\Lambda H^{\prime} B\right)_{{ji}}\frac{H^{2}_{{ij}}}{H^{\prime}_{{ij}}}, \end{array} $$

where, *Λ*,*B*,*H* are non-negative matrices, and *Λ*,*B* are symmetric matrices. And obviously the equality holds in Eq. 28 when *H*=*H* ^′^.

In Eq. 28, if we do the substitutions: *Λ*=*Y* ^*T*^ *Y*,*B*=*W* ^*T*^ *X* ^*T*^ *D* *X* *W*,*H*=*H*,*H* ^′^=*H* ^′^, we see that the fifth term of Eq. 27 is smaller than the fifth term of Eq. 26. However, the equality holds when *H*=*H* ^′^. Thus *G*(*H*,*H* ^′^) in Eq. 26 is an auxiliary function of *J*(*H*). □

Now, we need to find the global minimum of Eq. 26. Let *f*(*H*)=*G*(*H*,*H* ^′^). The gradient of *f*(*H*) is

29 $$\begin{array}{*{20}l} \frac{\partial f(H)}{\partial H_{{ij}}} = &-2\left(Y^{T}A^{T}DXW\right)_{{ij}}+2\lambda_{2}(QH)_{{ij}}\\ &+ 2\frac{\left(Y^{T}YH^{\prime}W^{T}X^{T}DXW\right)_{{ij}}H_{{ij}}}{{H^{\prime}}_{{ij}}} \end{array} $$

However, the second order derivative (i.e., the Hessian matrix) would be

30 $$\begin{array}{*{20}l} \frac{\partial^{2} f(H)}{\partial H_{{ij}}\partial H_{{kl}}} &= 2(Q)_{{jl}}\delta_{{ik}}\delta_{k\beta}&\\ & \quad+ \left(2\frac{\left(Y^{T}DYH^{\prime}W^{T}X^{T}XW\right)_{{ij}}}{H^{\prime}_{{ij}}} \right)\delta_{{jl}}\delta_{{ik}}& \end{array} $$

The Hessian matrix (Eq. 30 is semi-positive definite implying that *f*(*H*)=*G*(*H*,*H* ^′^) is a convex function. Thus, there exists a unique global minimum for *f*(*H*). The global minimum can be obtained by setting the gradient of *f*(*H*) to zero and solve for *H*. Thus from Eq. 29 we get

31 $$\begin{array}{@{}rcl@{}} H_{{ij}} = H^{\prime}_{{ij}}\frac{\left(Y^{T}A^{T}DXW\right)_{{ij}}}{\left(Y^{T}YH^{\prime}W^{T}X^{T}DXW + \lambda_{2} QH\right)_{{ij}}} \end{array} $$

By replacing *H* ^(*t*+1)^=*H* and *H* ^(*t*)^=*H* ^′^, we would obtain the *H* update rule in Algorithm 3. Therefore, under this rule, the objective function *J*(*H*) of Eq. 25 decreases monotonically, and hence completes the proof.

### Proof of Lemma 5

#### *Proof*

We know that,

$$\begin{array}{*{20}l} &tr\left(A-XW{H^{(t)}}^{T}Y^{T}\right)^{T}D\left(A-XW{H^{(t)}}^{T}Y^{T}\right)&\\ &\quad +\lambda_{1} tr\left(W^{T}PW\right) + \lambda_{2} tr\left({H^{(t)}}^{T}QH^{(t)}\right)&\\ &=\sum_{i=1}^{M}\sum_{j=1}^{N}\left(A-XW{H^{(t)}}^{T}Y^{T}\right)_{{ij}}D_{{ii}}&\\ &\quad +\lambda_{1} tr\left(W^{T}PW\right)+\lambda_{2}\sum_{k=1}^{n}\sum_{l=1}^{r} {H^{(t)}_{{kl}}}^{2}Q_{{kk}}&\\ &=\sum_{i=1}^{M}\left\|A_{i} - \left(XW{H^{(t)}}^{T}Y^{T}\right)_{i}\right\|^{2}D_{{ii}}&\\ &\quad +\lambda_{1} tr(W^{T}PW) + \lambda_{2} \sum_{k=1}^{n}\left\|H^{(t)}_{k}\right\|^{2} Q_{{kk}} \end{array} $$

Similarly, we can see that

$$\begin{array}{*{20}l} &tr\left(A-XW{H^{(t+1)}}^{T}Y^{T}\right)^{T}D\left(A-XW{H^{(t+1)}}^{T}Y^{T}\right)&\\ &\quad +\lambda_{1} tr\left(W^{T}PW\right) + \lambda_{2} tr\left({H^{(t+1)}}^{T}QH^{(t+1)}\right)&\\ &=\sum_{i=1}^{M}\left\|A_{i} - \left(XW{H^{(t+1)}}^{T}Y^{T}\right)_{i}\right\|^{2}D_{{ii}}&\\ &\quad +\lambda_{1} tr\left(W^{T}PW\right) + \lambda_{2} \sum_{k=1}^{n}\left\|H^{(t+1)}_{k}\right\|^{2} Q_{{kk}} \end{array} $$

Then, the right-hand side (*r*.*h*.*s*) of Eq. 21 becomes

$$\begin{array}{*{20}l} {}r.h.s &= \frac{1}{2}\sum_{i=1}^{M}\left(\left\|A_{i}-\left(XW{H^{(t+1)}}^{T}Y^{T}\right)_{i}\right\|^{2}\right.&\\ &\quad \left. -\left\|A_{i}-\left(\!XW{H^{(t)}}^{T}Y^{T}\!\right)_{i}\right\|\right)D_{{ii}} + \lambda_{2} \sum_{k=1}^{n}\left(\! \left\|H^{(t+1)}_{k}\right\|^{2} \right.&\\ &\quad \left.- \|H^{(t)}_{k}\|^{2}\right)Q_{{kk}}&\\ &=\frac{1}{2}\sum_{i=1}^{M}\left(\left\|A_{i}-\left(XW{H^{(t+1)}}^{T}Y^{T}\right)_{i}\right\|^{2} D_{{ii}} - \frac{1}{D_{{ii}}}\right)&\\ &\quad +\lambda_{2} \sum_{k=1}^{n}\left(\left\|H^{(t+1)}_{k}\right\|^{2}Q_{{kk}} - \frac{1}{Q_{{kk}}}\right)& \end{array} $$

And, the left-hand side (*l*.*h*.*s*) of Eq. 21 becomes

$$\begin{array}{*{20}l} l.h.s &=\sum_{i=1}^{M}\left(\sqrt{\left\|A_{i}-\left(XW{H^{(t+1)}}^{T}Y^{T}\right)_{i}\right\|^{2} }\right.&\\ &\quad \left. - \sqrt{\left\|A_{i}-\left(XW{H^{(t)}}^{T}Y^{T}\right)_{i}\right\|^{2}}\right)&\\ &\quad +\lambda_{2}\sum_{k=1}^{n}\left(\sqrt{\left\|H^{(t+1)}_{k}\right\|^{2}}-\sqrt{\left\|H^{(t)}_{k}\right\|^{2}}\right)&\\ &=\sum_{i=1}^{M}\left(\left\|A_{i}-\left(XW{H^{(t+1)}}^{T}Y^{T}\right)_{i}\right\| \right.&\\ &\quad \left.-\left\|A_{i}-\left(XW{H^{(t)}}^{T}Y^{T}\right)_{i}\right\|\right)&\\ &\quad +\lambda_{2}\sum_{k=1}^{n}\left(\sqrt{\left\|H^{(t+1)}_{k}\right\|^{2}}-\sqrt{\left\|H^{(t)}_{k}\right\|^{2}}\right)&\\ &= \sum_{i=1}^{M}\left(\left\|A_{i}-\left(XW{H^{(t+1)}}^{T}Y^{T}\right)_{i}\right\| - \frac{1}{D_{{ii}}}\right)&\\ &\quad +\lambda_{2} \left(\sum_{k=1}^{n}\left\|H^{(t+1)}_{k}\right\| - \frac{1}{Q_{{kk}}}\right)& \end{array} $$

Now, we compute the difference between the *l*.*h*.*s* and *r*.*h*.*s*,

$$\begin{array}{*{20}l} &l.h.s - r.h.s = \sum_{i=1}^{M}\left(\left\|A_{i}-\left(XW{H^{(t+1)}}^{T}Y^{T}\right)_{i}\right\|\right.&\\ &\quad \left.-\left\|A_{i}-\left(XW{H^{(t+1)}}^{T}Y^{T}\right)_{i}\right\|^{2}\frac{D_{{ii}}}{2} - \frac{1}{2D_{{ii}}}\right)&\\ &\quad +\lambda_{2}\sum_{k=1}^{n}\left(\left\|H^{(t+1)}_{k}\right\|-\left\|H^{(t+1)}_{k}\right\|^{2}\frac{Q_{{kk}}}{2}-\frac{1}{2Q_{{kk}}}\right)&\\ &=\sum_{i=1}^{M}\frac{D_{{ii}}}{2}\left(\frac{\left\|A_{i}-\left(XW{H^{(t+1)}}^{T}Y^{T}\right)_{i}\right\|}{D_{{ii}}}\right.&\\ &\quad \left.-\left\|A_{i}-\left(XW{H^{(t+1)}}^{T}Y^{T}\right)_{i}\right\|^{2}-\frac{1}{D^{2}_{{ii}}}\right)&\\ &\quad +\lambda_{2}\sum_{k=1}^{n}\frac{Q_{{kk}}}{2}\left(\frac{\left\|H^{(t+1)}_{k}\right\|}{Q_{{kk}}}-\left\|H^{(t+1)}_{k}\right\|^{2}-\frac{1}{Q^{2}_{{kk}}}\right)&\\ &=\sum_{i=1}^{M}\frac{(-D_{{ii}})}{2}\left(\left\|A_{i}-\left(XW{H^{(t+1)}}^{T}Y^{T}\right)_{i}\right\|-\frac{1}{D_{{ii}}} \right)^{2}&\\ &\quad +\lambda_{2}\sum_{k=1}^{n}\frac{(-Q_{{kk}})}{2}\left(\left\|H^{(t+1)}_{k}\right\|-\frac{1}{Q_{{kk}}}\right)^{2}&\\ &\leq 0&\end{array} $$

The above inequality holds because, *D*,*Q* are non-negative matrices, and the sum of non-positive numbers is always non-positive. This completes the proof. □

### Proof of Lemma 6

#### *Proof*

We can re-write Eq. 22 as follows:

32 $$\begin{array}{@{}rcl@{}} J(W^{(t+1)}) \leq J(W^{(t)}), \end{array} $$

where

33 $$\begin{array}{@{}rcl@{}}  J(W) &=& tr\left(A-XWH^{T}Y^{T}\right)^{T}D\left(A-XWH^{T}Y^{T}\right)\\ &&+\lambda_{1} tr\left(W^{T}PW\right) +\lambda_{2} tr\left(H^{T}QH\right) \end{array} $$

And, according to the statement of Lemma 6, under the *W* update rule in Algorithm 3, *J*(*W*) monotonically decreases. In order to prove the statement, we follow the approaches utilizing auxiliary functions [13, 14]. □

#### **Definition 2**

*G*(*W*,*W* ^′^) is an auxiliary function for the function *J*(*W*) if *G*(*W*,*W* ^′^)≥*J*(*W*) for all *W* ^′^ and *G*(*W*,*W*)=*J*(*W*).

Now, we define:

$$\begin{array}{@{}rcl@{}}  W^{(t+1)} = \underset{W}{\text{argmin}} \quad G\left(W,W^{(t)}\right) \end{array} $$

So, we have

$$\begin{array}{@{}rcl@{}} J\left(W^{(t+1)}\right) &=& G\left(W^{(t+1)},W^{(t+1)}\right) \leq G\left(W^{(t+1)},W^{(t)}\right)\\ &\leq & G\left(W^{(t)},W^{(t)}\right) = J\left(W^{(t)}\right) \end{array} $$

This proves that *J*(*W* ^(*t*)^) is monotonically decreasing.

Now the important steps in the remainder of the proof are: (a) determine a proper auxiliary function, and (b) find the global minima of the auxiliary function.

#### **Lemma 9**

The function

34 $$\begin{array}{*{20}l}  G(W,W^{\prime}) &= tr\left(A^{T}DA\right) - 2tr\left(YHW^{T}X^{T}DA\right)\\ &\quad +\lambda_{1} tr\left(W^{T}PW\right)+ \lambda_{2} tr\left(H^{T}QH\right) \\ &\quad + \sum_{i=1}^{m}\sum_{j=1}^{r} \frac{\left(X^{T}DXW^{\prime}H^{T}Y^{T}YH\right)_{{ij}}W^{2}_{{ij}}}{{W^{\prime}}_{{ij}}}  \end{array} $$

is an auxiliary function for *J*.

#### *Proof*

Now *J*(*W*) of Eq. 41 can be re-written as:

35 $$\begin{array}{*{20}l} J(W) &= tr\left(A^{T}DA\right) - 2tr\left(YHW^{T}X^{T}DA\right)\\ &\quad + \lambda_{1} tr\left(W^{T}PW\right)+ \lambda_{2} tr\left(H^{T}QH\right)\\ &\quad + tr\left(W^{T}X^{T}DXWH^{T}Y^{T}YH\right) \end{array} $$

Now we will be applying the following inequality of matrices according to the investigations by [14, 15]:

36 $$\begin{array}{@{}rcl@{}} tr\left(W^{T}\Lambda WB\right) \leq \sum_{i}\sum_{j}\left(\Lambda W^{\prime} B\right)_{{ji}}\frac{W^{2}_{{ij}}}{W^{\prime}_{{ij}}}, \end{array} $$

where, *Λ*,*B*,*W* are non-negative matrices, and *Λ*,*B* are symmetric matrices. And obviously the equality holds in Eq. 36 when *W*=*W* ^′^.

In Eq. 36, if we do the substitutions: *Λ*=*X* ^*T*^ *D* *X*,*B*=*H* ^*T*^ *Y* ^*T*^ *Y* *H*,*W*=*W*,*W* ^′^=*W* ^′^, we see that the fifth term of Eq. 35 is smaller than the fifth term of Eq. 34. However, the equality holds when *W*=*W* ^′^. Thus *G*(*W*,*W* ^′^) in Eq. 34 is an auxiliary function of *J*(*W*). □

Now, we need to find the global minimum of Eq. 34. Let *f*(*W*)=*G*(*W*,*W* ^′^). The gradient of *f*(*W*) is

37 $$\begin{array}{*{20}l} \frac{\partial f(W)}{\partial W_{{ij}}} = &-2(X^{T}DAYH)_{{ij}}+2\lambda_{1}(PW)_{{ij}}\\ &+ 2\frac{\left(X^{T}DXW^{\prime}H^{T}Y^{T}YH\right)_{{ij}}W_{{ij}}}{{W^{\prime}}_{{ij}}} \end{array} $$

However, the second order derivative (i.e., the Hessian matrix) would be

38 $$\begin{array}{*{20}l} \frac{\partial^{2} f(W)}{\partial W_{{ij}}\partial W_{{kl}}} &= 2(P)_{{ij}}\delta_{{jl}}\delta_{{ik}}&\\ &\quad + \left(2\frac{\left(X^{T}DXW^{\prime}H^{T}Y^{T}YH\right)_{{ij}}}{W^{\prime}_{{ij}}} \right)\delta_{{jl}}\delta_{{ik}}& \end{array} $$

The Hessian matrix (Eq. 38) is semi-positive definite implying that *f*(*W*)=*G*(*W*,*W* ^′^) is a convex function. Thus, there exists a unique global minimum for *f*(*W*). The global minimum can be obtained by setting the gradient of *f*(*W*) to zero and solve for *W*. Thus from Eq. 37 we get

39 $$\begin{array}{@{}rcl@{}} W_{{ij}} = W^{\prime}_{{ij}}\frac{\left(X^{T}DAYH\right)_{{ij}}}{\left(X^{T}DXW^{\prime}H^{T}Y^{T}YH + \lambda_{1} PW\right)_{{ij}}} \end{array} $$

By replacing *W* ^(*t*+1)^=*W* and *W* ^(*t*)^=*W* ^′^, we would obtain the *W* update rule in Algorithm 3. Therefore, under this rule, the objective function *J*(*W*) of Eq. 41 decreases monotonically, and hence completes the proof.

### Proof of Lemma 7

#### *Proof*

We know that,

$$\begin{array}{*{20}l} &tr\left(A-XW^{(t)}{H}^{T}Y^{T}\right)^{T}D\left(A-XW^{(t)}{H}^{T}Y^{T}\right)&\\ &\quad +\lambda_{1} tr\left({W^{(t)}}^{T}PW^{(t)}\right) + \lambda_{2} tr\left({H}^{T}QH\right)&\\ &=\sum_{i=1}^{M}\sum_{j=1}^{N}\left(A-XW^{(t)}{H}^{T}Y^{T}\right)_{{ij}}D_{{ii}}&\\ &\quad +\lambda_{1} \sum_{k=1}^{m}\sum_{l=1}^{r} {W^{(t)}_{{kl}}}^{2}P_{{kk}}+\lambda_{2} tr\left(H^{T}QH\right)&\\ &=\sum_{i=1}^{M}\left\|A_{i} - \left(XW^{(t)}{H}^{T}Y^{T}\right)_{i}\right\|^{2}D_{{ii}}&\\ &\quad +\lambda_{1} \sum_{k=1}^{m}\left\|W^{(t)}_{k}\right\|^{2} P_{{kk}} + \lambda_{2} tr(H^{T}QH)& \end{array} $$

Similarly, we can see that

$$\begin{array}{*{20}l} &tr\left(A-XW^{(t+1)}{H}^{T}Y^{T}\right)^{T}D\left(A-XW^{(t+1)}{H}^{T}Y^{T}\right)&\\ &\quad +\lambda_{1} tr\left({W^{(t+1)}}^{T}PW^{(t+1)}\right) + \lambda_{2} tr\left({H}^{T}QH\right)&\\ &=\sum_{i=1}^{M}\left\|A_{i} - \left(XW^{(t+1)}{H}^{T}Y^{T}\right)_{i}\right\|^{2}D_{{ii}}&\\ &\quad +\lambda_{1} \sum_{k=1}^{m}\left\|W^{(t+1)}_{k}\right\|^{2} P_{{kk}} + \lambda_{2} tr(H^{T}QH) \end{array} $$

Then, the right-hand side (*r*.*h*.*s*) of Eq. 23 becomes

$${}\begin{aligned} r.h.s &= \frac{1}{2}\sum_{i=1}^{M}\left(\left\|A_{i}-\left(XW^{(t+1)}{H}^{T}Y^{T}\right)_{i}\right\|^{2}\right.&\\ &\quad \left. -\left\|A_{i}\,-\,\left(\! XW^{(t)}{H}^{T}Y^{T} \!\right)_{i} \!\right\|\right)D_{{ii}} \,+\, \lambda_{1} \sum_{k=1}^{m}\left(\! \left\|W^{(t+1)}_{k}\right\|^{2} \right.&\\ &\quad \left.- \left\|W^{(t)}_{k}\right\|^{2}\right)P_{{kk}}&\\ &=\frac{1}{2}\sum_{i=1}^{M}\left(\left\|A_{i}-\left(XW^{(t+1)}{H}^{T}Y^{T}\right)_{i}\right\|^{2} D_{{ii}} - \frac{1}{D_{{ii}}}\right)&\\ &\quad +\lambda_{1} \sum_{k=1}^{m}\left(\left\|W^{(t+1)}_{k}\right\|^{2}P_{{kk}} - \frac{1}{P_{{kk}}}\right)& \end{aligned} $$

And, the left-hand side (*l*.*h*.*s*) of Eq. 23 becomes

$$\begin{array}{*{20}l} l.h.s & =\sum_{i=1}^{M}\left(\sqrt{\left\|A_{i}-\left(XW^{(t+1)}{H}^{T}Y^{T}\right)_{i}\right\|^{2} }\right.&\\ & \quad \left. - \sqrt{\left\|A_{i}-\left(XW^{(t)}{H}^{T}Y^{T}\right)_{i}\right\|^{2}}\right)&\\ & \quad +\lambda_{1}\sum_{k=1}^{m}\left(\sqrt{\left\|W^{(t+1)}_{k}\right\|^{2}}-\sqrt{\left\|W^{(t)}_{k}\right\|^{2}}\right)&\\ &=\sum_{i=1}^{M}\left(\left\|A_{i}-\left(XW^{(t+1)}{H}^{T}Y^{T}\right)_{i}\right\| \right.&\\ &\quad \left. - \left\|A_{i}-\left(XW^{(t)}{H}^{T}Y^{T}\right)_{i}\right\|\right)&\\ &\quad + \lambda_{1}\sum_{k=1}^{m}\left(\sqrt{\left\|W^{(t+1)}_{k}\right\|^{2}}-\sqrt{\left\|W^{(t)}_{k}\right\|^{2}}\right)&\\ &= \sum_{i=1}^{M}\left(\left\|A_{i}-\left(XW^{(t+1)}{H}^{T}Y^{T}\right)_{i}\right\| - \frac{1}{D_{{ii}}}\right)&\\ & \quad + \lambda_{1} \left(\sum_{k=1}^{m}\left\|W^{(t+1)}_{k}\right\| - \frac{1}{P_{{kk}}}\right)& \end{array} $$

Now, we compute the difference between the *l*.*h*.*s* and *r*.*h*.*s*,

$${}\begin{aligned} l.h.s - r.h.s &= \sum_{i=1}^{M}\left(\left\|A_{i}-\left(XW^{(t+1)}{H}^{T}Y^{T}\right)_{i}\right\|\right.&\\ & \quad \left.-\left\|A_{i}-\left(XW^{(t+1)}{H}^{T}Y^{T}\right)_{i}\right\|^{2}\frac{D_{{ii}}}{2} - \frac{1}{2D_{{ii}}}\right)&\\ & \quad +\! \lambda_{1}\!\sum_{k=1}^{m}\!\left(\! \left\|W^{(t+1)}_{k}\right\|\,-\,\left\|W^{(t+1)}_{k}\right\|^{2}\!\frac{P_{{kk}}}{2}\,-\,\frac{1}{2P_{{kk}}}\!\right)&\\ &=\sum_{i=1}^{M}\frac{D_{{ii}}}{2}\left(\frac{\left\|A_{i}-\left(XW^{(t+1)}{H}^{T}Y^{T}\right)_{i}\right\|}{D_{{ii}}}\right.&\\ & \quad \left.-\left\|A_{i}-\left(XW^{(t+1)}{H}^{T}Y^{T}\right)_{i}\right\|^{2}-\frac{1}{D^{2}_{{ii}}}\right)&\\ & \quad + \lambda_{1}\!\sum_{k=1}^{m}\!\frac{P_{{kk}}}{2}\!\left(\! \frac{\left\|W^{(t+1)}_{k}\right\|}{P_{{kk}}}\,-\,\left\|W^{(t+1)}_{k}\right\|^{2}\,-\,\frac{1}{P^{2}_{{kk}}}\!\right)&\\ & =\! \sum_{i=1}^{M}\!\frac{(\! -D_{{ii}})}{2}\!\left(\!\left\|A_{i}\,-\,\left(\! XW^{(t+1)}{H}^{T}Y^{T}\!\right)_{i}\right\|\,-\,\frac{1}{D_{{ii}}} \!\right)^{\!2}&\\ & \quad +\lambda_{1}\sum_{k=1}^{m}\frac{(-P_{{kk}})}{2}\left(\left\|W^{(t+1)}_{k}\right\|-\frac{1}{P_{{kk}}}\right)^{2}&\\ & \quad \leq 0& \end{aligned} $$

The above inequality holds because, *D*,*P* are non-negative matrices, and the sum of non-positive numbers is always non-positive. This completes the proof. □

### Correctness of the RIMC Algorithm (version 2)

In this section we are going to prove that the converged solution presented in Algorithm 3 is the correct optimal solution. In fact, we will show that the converged solution satisfies the Karush-Kuhn-Tucker (KKT) condition of the constrained optimization theory. At first, we have Theorem 10 to prove the correctness of the algorithm with respect to *W*. Theorem 11 will prove the correctness of the algorithm with respect to *H*.

#### **Theorem 10**

At convergence, the converged solution *W* ^∗^ of the updating rule in Algorithm *3* satisfies the KKT condition.

#### *Proof*

The KKT condition for *W* with constraints *W*_*αβ*_≥0, with *α*=1,⋯,*m*;*β*=1,⋯,*r* is:

40 $$\begin{array}{@{}rcl@{}} \frac{\partial J(W)}{\partial W_{\alpha\beta}}W_{\alpha\beta} = 0, \forall \alpha,\beta \end{array} $$

Similar to Eq. 25, the *J*(*W*) can be written as:

41 $$\begin{array}{@{}rcl@{}} J(W) &=& tr\left(A-XWH^{T}Y^{T}\right)^{T}D\left(A-XWH^{T}Y^{T}\right)\\ &&+\lambda_{1} tr\left(W^{T}PW\right) +\lambda_{2} tr\left(H^{T}QH\right) \end{array} $$

Now, the partial derivative of *J*(*W*) can be expressed as:

42 $$\begin{array}{*{20}l} \frac{\partial J(W)}{\partial W_{\alpha\beta}} = &-2\left(X^{T}DAYH\right)_{\alpha\beta}+2\lambda_{1}(PW)_{\alpha\beta}&\\ &+2\left(X^{T}DXWH^{T}Y^{T}YH\right)_{\alpha\beta}&\end{array} $$

Thus, the KKT condition for *W* is:

43 $$\begin{array}{*{20}l}  &\left[-\left(X^{T}DAYH\right)_{\alpha\beta} + \lambda_{1}(PW)_{\alpha\beta}\right.&\\ &\;\;+\left. \left(X^{T}DXWH^{T}Y^{T}YH\right)_{\alpha\beta}\right]W_{\alpha\beta} =0, \forall \alpha,\beta &  \end{array} $$

But, once *W* converges (according to Algorithm 3), the converged solution *W* ^∗^ satisfies the following:

$$\begin{array}{*{20}l} &W^{*}_{\alpha\beta} \leftarrow W^{*}_{\alpha\beta}\frac{\left(X^{T}DAYH\right)_{\alpha\beta}}{\left(X^{T}DXW^{*}H^{T}Y^{T}YH+\lambda_{1}PW^{*}\right)_{\alpha\beta}}& \end{array} $$

which can be written as

44 $$\begin{array}{*{20}l}  &\left[-\left(X^{T}DAYH\right)_{\alpha\beta} + \lambda_{1}(PW^{*})_{\alpha\beta}\right.&\\ &\;\;+\left. \left(X^{T}DXW^{*}H^{T}Y^{T}YH\right)_{\alpha\beta}\right]W^{*}_{\alpha\beta} =0, \forall \alpha,\beta &\end{array} $$

This is identical to Eq. 43. Thus, the converged solution *W* ^∗^ satisfies the KKT condition. □

#### **Theorem 11**

At convergence, the converged solution *H* ^∗^ of the updating rule in Algorithm *3* satisfies the KKT condition.

#### *Proof*

The KKT condition for *H* with constraints *H*_*γψ*_≥0, with *γ*=1,⋯,*n*,*ψ*=1,⋯,*r* is:

45 $$\begin{array}{@{}rcl@{}} \frac{\partial J(H)}{\partial H_{\gamma\psi}}H_{\gamma\psi} = 0, \forall \gamma,\psi \end{array} $$

Now, the partial derivative of *J*(*H*) from Eq. 25 is

46 $$\begin{array}{*{20}l}  \frac{\partial J(H)}{\partial H_{\gamma\psi}} = &-2\left(Y^{T}A^{T}DXW\right)_{\gamma\psi} + 2\lambda_{2}(QH)_{\gamma\psi}&\\ &+2\left(Y^{T}YHW^{T}X^{T}DXW\right)_{\gamma\psi}& \end{array} $$

Thus, the KKT condition for *H* is:

47 $$\begin{array}{*{20}l}  &\left[-\left(Y^{T}A^{T}DXW\right)_{\gamma\psi} + \lambda_{2}(QH)_{\gamma\psi}\right.&\\ &\;\; +\left. \left(Y^{T}YHW^{T}X^{T}DXW\right)_{\gamma\psi}\right]H_{\gamma\psi} =0, \forall \gamma,\psi &  \end{array} $$

But, once *H* converges (according to Algorithm 3), the converged solution, *H* ^∗^ satisfies the following:

$$\begin{array}{*{20}l}  &H^{*}_{\gamma\psi} \leftarrow H^{*}_{\gamma\psi}\frac{\left(Y^{T}A^{T}DXW\right)_{\gamma\psi}}{\left(Y^{T}YH^{*}W^{T}X^{T}DXW+\lambda_{2}QH^{*}\right)_{\gamma\psi}}&\end{array} $$

which can be written as

$$\begin{array}{*{20}l}  &\left[-\left(Y^{T}A^{T}DXW\right)_{\gamma\psi} + \lambda_{2}(QH^{*})_{\gamma\psi}\right.&\\  &\;\;+ \left. \left(Y^{T}YH^{*}W^{T}X^{T}DXW\right)_{\gamma\psi}\right]H^{*}_{\gamma\psi} =0, \forall \gamma,\psi&\end{array} $$

This is identical to Eq. 47. Thus, the converged solution *H* ^∗^ satisfies the KKT condition. □

### Stable robust IMC (SRIMC) formulation

Instead of solving the RIMC objective function (Eq. 7) directly, here we propose a two-step solution strategy to the RIMC formulation, and we call this new algorithm SRIMC.

#### Step 1: solving matrix *Z* from a matrix equation

In this step, we consider the following matrix equation

48 $$\begin{array}{*{20}l} XZY^{T} = A, \end{array} $$

where *Z* is an *m*×*n* matrix of unknowns, *X* is the *M*×*m* feature matrix of the row entities, *Y* is the *N*×*n* is the feature matrix of the column entities. And, *A* is the *M*×*N* binary association matrix between the row and column entities.

Now, in Eq. 48, if we left multiply by *X* ^*T*^ and right multiply by *Y*, we get the following equation

49 $$\begin{array}{@{}rcl@{}} X^{T}XZY^{T}Y = X^{T}AY \end{array} $$

If *X* has full column rank and *Y* has a full row rank, then both *X* ^*T*^ *X* and *Y* ^*T*^ *Y* are invertible. Therefore, we can solve for *Z*.

50 $$\begin{array}{@{}rcl@{}}  ZY^{T}Y &=& \left(X^{T}X\right)^{-1}X^{T}AY\\ \Rightarrow \hat{Z} &=& \left(X^{T}X\right)^{-1}X^{T}AY\left(Y^{T}Y\right)^{-1} \end{array} $$

#### Step 2: robust NMF on matrix *Z*

51 $$\begin{array}{*{20}l}  \underset{W,H}{\text{min}} &\quad\varphi = \|Z-WH^{T}\|_{2,1} + \lambda_{1} \|W\|_{2,1} + \lambda_{2} \|H\|_{2,1}&\\ &\text{such that},\quad W\geq 0, H\geq 0& \end{array} $$

This a modified non-negative matrix factorization (NMF) problem; only difference is the usage of the *ℓ*_2,1_ norms instead of *ℓ*_2_ norms in the loss function and the regularizers.

### Algorithm for SRIMC

We can also solve the Stable Robust IMC optimization problem by solving the two problems mentioned above. It is demonstrated in Algorithm 4.



## Results

### Disease-LincRNA association datasets

We prepared a sparse association matrix by extracting the lincRNA-disease association dataset from the LncRNADisease [4] with sparsity indx 0.22%. LincRNA expression dataset was obtained from the co-expression based association study [7]. Finally, we cataloged 8194 lincRNAs and 2148 human disease phenotypes and the resulting association matrix contains 46,934 associations among these two entities. We followed a standard naming of the disease phenotypes by OMIM identification numbers. We extracted top-5 OMIM phenotypes matching the human disease names using OMIM API [16].

### LincRNA feature datasets

The features of LincRNAs consist of four groups of information: (i) expression profiles, (ii) transcriptor factor binding sites (TFBS), (iii) functional annotations and (iv) single nucleotide polymorphism (SNP) information. The RNA-seq expression profiles of the 8194 lincRNAs on 22 human tissues were collected from the Human BodyMap Project 2.0 [3]. The expression scores were measured in FPKM (Fragments Per Kilobase of exons per Million Fragments mapped) unit. Then, TFBS information about the lincRNAs in our study with 120 transcription factors were obtained from ChIP-base dataset [17]. Linc2GO is a public data repository containing functional annotations of lincRNAs [18]. There are three different types of functions cataloged in the Lin2GO dataset: gene ontology biological process (GO BP), gene ontology molecular function (GO MF) and KEGG pathways. The 8194 lincRNAs with the functional annotation together make a sparse matrix with sparsity index 0.11%. We performed singular value decomposition on the matrix to compute and use the leading 100 singular vectors in our study as part of the features of the lincRNAs. We extracted links among 368,494 SNPs and the lincRNAs from our study from the lncRNASNP dataset [19]. Again, the SNP-lincRNA association matrix turned out to a sparse matrix with the sparsity index 0.0077%. Therefore, we performed singular value decomposition on the matrix to compute and use the leading 100 singular vectors. Finally, we performed a filtering on all the four groups of features of the lincRNAs in our study. We found that 6540 out of the initial 8194 lincRNAs have data from all the four groups of featureset. Therefore, our final lincRNA feature matrix (*X* in our study) has 6540 rows (lincRNAs) and 342 columns (features).

### Disease feature datasets

The disease feature dataset consists of two groups of information: (i) term frequency inverse document frequency (TF-IDF) scores and (ii) phenotype similarity scores. The TFIDF scores were prepared by mining the OMIM text corpus on the 2661 OMIM phenotypes, resulting a 20491 term scores of each of the 2148 phenotypes from our study. We took leading 100 singular vectors as part of the disease feature. The phenotype-phenotype similarity scores were retrieved from a study conducted by [20]. The similarity profiles after encapsulated in a square matrix of dimension 2148 by 2148, had to go through a singular value decomposition module to extract leading 100 singular vectors that constitute the part of the feature matrix of the diseases in our study. Finally, our disease feature matrix contains 200 features of the 2148 diseas es.

### Baseline algorithms

We conducted a comparative study of our proposed algorithms with five baseline methods: (i) NMF [13], (ii) LRLSLDA [5], (iii) TsLincRNA-Disease [7], (iv) K-RWRH [6] and (v) standard IMC [21]. The NMF based approach finds the two factors *W* and *H* by just working on the lincRNA-disease association matrix *A*. The LRLSLDA ranks the lincRNAs with a disease by the use of a classifier trained on two similarity feature matrices. The method was developed with eight parameters to train before getting good prediction results. The TsLincRNA-Disease utilizes a series of statistical significance tests on a co-expression network obtained from tissue-specific and non-tissue-specific lincRNA expression information. Apart from the expression data, this method lacks the integration of other types of information available about the lincRNAs and the disease. The K-RWRH is a stochastic algorithm developed on top of the random walk on a three heterogeneous networks. The method is very complex and it is harder to obtain a steady state distribution for the dataset our study.

### Evaluation metrics

We define two metrics for evaluating our proposed algorithm and the baseline algorithms. The metrics are popular in evaluating any recommender style systems as in [22].

**p** **r** **e** **c** **i** **s** **i** **o** **n** **@** **k**: The ratio of the number of recovered disease phenotypes to recommended *k* phenotypes for a target lincRNA. We take average of the ratios for every lincRNAs of our study. The metric is defined as follows:

52 $$\begin{array}{@{}rcl@{}} precision@k = \frac{1}{N_{l}}\sum_{l=1}^{N_{l}}\frac{|P_{l}(k) \cap D_{l}|}{k}, \end{array} $$

where, *P*_*l*_(*k*) is the top-*k* ranked diseases for an lincRNA *l*, *D*_*l*_ is the set of diseases related to the lincRNA *l* deleted during the training phase. And, *N*_*l*_ is the total number lincRNAs in the test set.

**r** **e** **c** **a** **l** **l** **@** **k**: The ratio of recovered disease phenotypes to the set of hidden phenotypes in the test dataset. Again, we take average of the ratios for every lincRNAs in the study. The metric is defined as follows:

53 $$\begin{array}{@{}rcl@{}} recall@k = \frac{1}{N_{l}}\sum_{l=1}^{N_{l}}\frac{|P_{l}(k) \cap D_{l}|}{|D_{l}|}, \end{array} $$

We repeated the experiments for various values of *k*, from 5 to 100. We conducted 10-fold cross-validation in each of the experiments listed in the following sections.

## Discussions

### True LincRNA-disease association retrieval

**Fig. 1:**
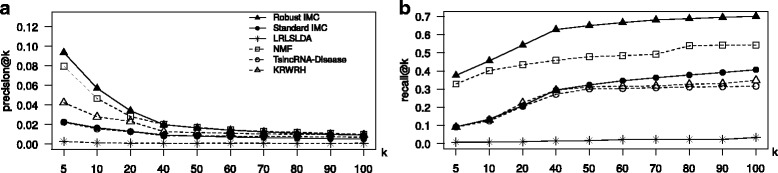
Comparision of lincRNA-disease association methods. **a** *k*-vs- *p* *r* *e* *c* *i* *s* *i* *o* *n* *@* *k* plot for all the six methods. **b** *k*-vs- *r* *e* *c* *a* *l* *l* *@* *k* plot for the six methods. The standard IMC and the proposed RIMC method is trained with 342 lincRNA features and 200 disease features, with a rank, *r*=100. NMF was trained with the same binary association matrix we used in the IMC experiments with a rank *r*=100

Figure 1 shows the performance of RIMC along with other base-line algorithms to predict true lincRNA-disease associations. A 10-fold cross-validation was conducted on the 2418 OMIM phenotypes. We find that our RIMC method leads in identifying true associations than all the baseline algorithms for all *k* values. The NMF based algorithm is better than the three other baseline algorithms. LRLSLDA’s association retrieval was the worse due to the fact that it relies only on known association matrix and the expression profiles of the lincRNAs that seems to be not sufficient to build one predictive model.

### Induction on new associations

Here we conducted a thorough comparative study on the three algorithms including two of ours (RIMC and SRIMC) to predict associations between novel lincRNAs and/or diseases. We assume that all the features of the novel lincRNAs and/or diseases that we bring into our prediction framework can be computed or available. Note that,none of the baseline algorithms except the standard inductive matrix completion based approach (standard IMC) are missing in all the experiments from this sections due to the fact that none are capable of doing induction on novel associations.

#### Induction experiments on new LincRNAs

**Fig. 2:**
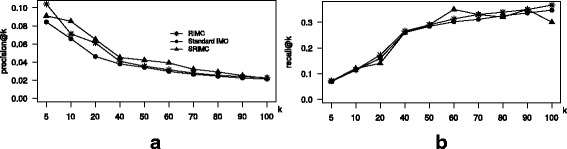
Performance comparison of the standard IMC, RIMC and SRIMC for induction on existing set of diseases and new lincRNAs. **a** *k*-vs- *p* *r* *e* *c* *i* *s* *i* *o* *n* *@* *k* plot for the two methods, **b** *k*-vs- *r* *e* *c* *a* *l* *l* *@* *k* plot for the two methods

From the dataset in our study we selected a list of 10% lincRNAs and deleted all the entries of these randomly selected lincRNAs from the three training matrices *A*,*X* and *Y*. The deleted entries will serve as test set during evaluation. Then, RIMC, SRIMC and the standard IMC were trained with modified training matrices. Once, training is done on the reduced dataset, each of the obtained three modules were evaluated with the test set that were extracted at the beginning of this step. We repeat the entire training and test steps 10 times and reported the average performance score of all the three methods. Figure 2 illustrates the performance comparison of the three methods for predicting association between a new lincRNA with an existing set of diseases. RIMC and SRIMC show better *p* *r* *e* *c* *i* *s* *i* *o* *n* *@* *k* than the standard IMC based approach for predicting upto the top-50 disease associations with the new lincRNAs. For higher values of *k* in the top-*k* predictions, both RIMC and the standard IMC show similar performance. But in terms of numerical precision, RIMC exceeds the performance of standard IMC. However, in terms of *r* *e* *c* *a* *l* *l* *@* *k*, we can see that SRIMC and RIMC perform superior than that of the standard IMC method.

#### Induction experiments on new diseases

**Fig. 3:**
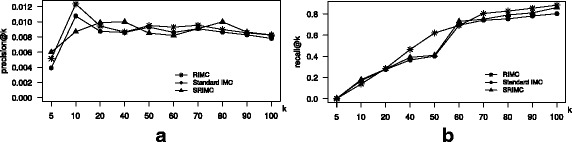
Performance comparison of the standard IMC, RIMC and SRIMC for induction on new diseases and existing set of lincRNAs. **a** *k*-vs- *p* *r* *e* *c* *i* *s* *i* *o* *n* *@* *k* plot for the two methods, **b** *k*-vs- *r* *e* *c* *a* *l* *l* *@* *k* plot for the two methods

Similar to the approach mentioned in the previous section, we randomly selected 10% of the total disease phenotypes from the dataset of the study, and deleted all the entries related to the diseases. The deleted entries is going to be our test set. The reduced dataset is going to serve as training dataset. The RIMC, SRIMC and the standard IMC were trained on the reduced training dataset and evaluated against the test set. The entire training and evaluation were repeated 10 times and the average performance scores were reported. Figure 3 illustrates the performance comparison of the three methods to predict associations among known list of lincRNAs with a novel disease. Here, both RIMC and SRIMC demonstrates better induction performance in terms of the *p* *r* *e* *c* *i* *s* *i* *o* *n* *@* *k* and *r* *e* *c* *a* *l* *l* *@* *k* values.

#### Induction experiments on both new LincRNAs and new diseases

**Fig. 4:**
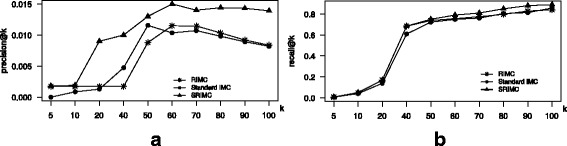
Performance comparison of the standard IMC, RIMC and SRIMC for induction on both new diseases and new lincRNAs. **a** *k*-vs- *p* *r* *e* *c* *i* *s* *i* *o* *n* *@* *k* plot for the two methods, **b** *k*-vs- *r* *e* *c* *a* *l* *l* *@* *k* plot for the two methods

Finally, in this batch of induction experiment, we randomly picked 5% of the subject disease entries, and 5% of the subject lincRNA entries and deleted the respective connections between the two entities from the three data matrices *A*,*X* and *Y*. The deleted connections and feature set are treated as the test-set, while the reduced data matrices are used to train the three algorithms. We repeat the above steps 10 times and compute the average performance scores. Figure 4 illustrates the performance comparison of our proposed RIMC, SRIMC and the only baseline algorithm applicable here which is the standard IMC to predict association between a new lincRNA and a new disease based on the model trained on data about a limited set of lincRNAs and disease phenotypes not including these two lincRNA and disease phenotypes. The *p* *r* *e* *c* *i* *s* *i* *o* *n* *@* *k* plot of for the RIMC and SRIMC show better performance than the standard IMC based approach for predicting for both lower and higher values of *k* in the top-*k* association ranking with the novel diseases. However, from the *r* *e* *c* *a* *l* *l* *@* *k* cure of the both algorithms, we can see that both RIMC and standard IMC performs similar in the top-*k* association prediction problem. But, SRIMC performs superior than both of the algorithms.

## Conclusions

In this article, we propose theoretical foundations of robust inductive matrix completion method using *ℓ*_2,1_ norm. We provided three algorithms to solve our robust induction matrix completion objective function. The first two algorithms are equivalent, but the third one what we call Stable Robust Inductive Matrix Completion (SRIMC) breaks the problem into two sub-problems. But it turns out to be a simple, stable and better solution strategy. We applied the proposed methods in identifying missing links between putative lincRNAs and human disease phenotypes. All the three variants of robust inductive matrix completion are well suited for noisy type of datasets. Besides the standard IMC formulation, our proposed method also outperformed other four lincRNA-disease association solutions. The proposed methods are applicable to predict associations among between well-studied lincRNAs with novel disease, or novel lincRNAs with well-studied diseases, or a set of novel lincRNAs with novel diseases.

## Abbreviations

FPKM: Fragments per kilobase of exons per million fragments
GWAS: Genome wide association studies
HTS: High throughput sequencing
IMC: Inductive matrix completion
lincRNAs: Long intergenic non-coding RNAs
lncRNAs: Long non-coding RNAs
NMF: Non-negative matrix factorization
RIMC: Robust inductive matrix completion
SRIMC: Stable robust inductive matrix completion
SNP: Single nucleotide polymorphism
TFBS: Transcriptor factor binding sites
TF-IDF: Term frequency inverse document frequency
